# The Multifaceted Impact of Gallstones: Understanding Complications and Management Strategies

**DOI:** 10.7759/cureus.62500

**Published:** 2024-06-16

**Authors:** Varsha Gupta, Abhinav Abhinav, Srineil Vuthaluru, Shivam Kalra, Akshita Bhalla, Abhinav K Rao, Manjeet K Goyal, Ashita R Vuthaluru

**Affiliations:** 1 Anesthesiology, Maharishi Markandeshwar Institute of Medical Sciences and Research, Ambala, IND; 2 Gastroenterology, Dayanand Medical College and Hospital, Ludhiana, IND; 3 General Surgery, University of Nebraska Medical Center, Omaha, USA; 4 Internal Medicine, Trident Medical Center, North Charleston, USA; 5 Internal Medicine, Dayanand Medical College and Hospital, Ludhiana, IND; 6 Internal Medicine, Punjab Institute of Medical Sciences, Jalandhar, IND; 7 Gastroenterology and Hepatology, Dayanand Medical College and Hospital, Ludhiana, IND; 8 Anesthesia and Critical Care, Maharishi Markandeshwar Institute of Medical Sciences and Research, Ambala, IND

**Keywords:** gallstone ileus, laparoscopic cholecystectomy in mirizzi syndrome, multiple choledocholithiasis, cholelithiasis, gall bladder stones

## Abstract

Gallstones, or cholelithiasis, represent a prevalent gastrointestinal disorder characterized by the formation of calculi within the gallbladder. This review aims to provide a comprehensive analysis of the complications associated with gallstones, with a focus on their pathophysiology, clinical manifestations, diagnostic methodologies, and management strategies. Gallstone-related complications encompass a broad spectrum, including biliary colic, acute cholecystitis, choledocholithiasis, acute pancreatitis, and cholangitis. The pathogenesis of these complications primarily involves biliary obstruction and subsequent infection, leading to significant morbidity and potential mortality. Diagnostic evaluation of gallstone complications employs various imaging techniques, such as ultrasonography, magnetic resonance cholangiopancreatography (MRCP), and endoscopic retrograde cholangiopancreatography (ERCP), each with distinct advantages and limitations. Therapeutic approaches are discussed, ranging from conservative management with pharmacotherapy and bile acid dissolution agents to interventional procedures like extracorporeal shock wave lithotripsy (ESWL) and percutaneous cholecystostomy. Surgical management, particularly laparoscopic cholecystectomy, remains the gold standard for definitive treatment. Additionally, advancements in endoscopic techniques, including endoscopic sphincterotomy (EST) and cholangioscopy, are highlighted. This review synthesizes current research findings and clinical guidelines, aiming to enhance the understanding and management of gallstone-related complications among healthcare professionals, thereby improving patient outcomes and reducing the burden of this common ailment.

## Introduction and background

**Figure 1:**
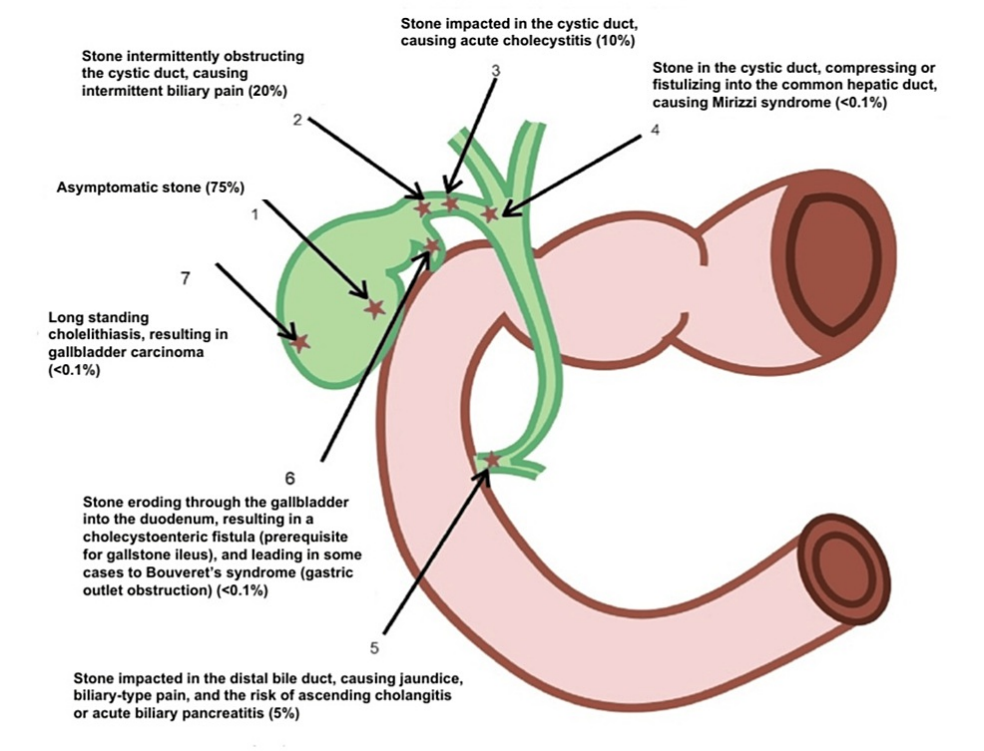
Complications of cholelithiasis The percentages indicate the incidence of complications. Image Credit: Author Goyal MK

Cholelithiasis is one of the most common gastrointestinal problems affecting around 20% of the global population. It is asymptomatic in the majority and only 20% of the patients develop symptoms related to it [1]. Diabetes mellitus and cholelithiasis are interrelated medical conditions with significant clinical implications. Diabetes mellitus, a metabolic disorder characterized by chronic hyperglycemia due to insulin deficiency or resistance, is associated with an increased risk of gallstone formation or cholelithiasis. This association is primarily attributed to the dysregulation of lipid metabolism and impaired biliary motility observed in diabetic patients. Cholelithiasis, the formation of gallstones within the gallbladder, often comprises cholesterol or bilirubin stones [1]. Epidemiological studies indicate a higher prevalence of gallstones among individuals with type 2 diabetes mellitus, which may exacerbate complications such as acute cholecystitis and pancreatitis. Elucidating the pathophysiological mechanisms linking diabetes mellitus and cholelithiasis is essential for developing effective preventive strategies and optimizing therapeutic interventions for patients affected [2]. The symptomatic disease causes a wide spectrum of manifestations of uncomplicated repeated pain attacks of complications like acute cholecystitis, common bile duct (CBD) obstruction, pancreatitis, and cholangitis. These complications significantly affect the quality of life while causing high healthcare costs. In this review, we will delve into various complications of cholelithiasis briefly excluding biliary pancreatitis and carcinoma of the gallbladder. Various complications are depicted in Figure 1.

Epidemiology

Cholelithiasis is one of the most common diseases in India, with an estimated prevalence of 2% to 29% as per various studies [2]. The disease is as much as five times more common in North India [3]. Dietary differences in the two regions are considered to be responsible for such a high prevalence in North India. The disease is more common in females attributable to more sedentary habits and ovarian hormones in them. The incidence of cholelithiasis increases with age, with the disease being around four to 10 times more common in the elderly age group. Acute cholecystitis is the most common complication of gallstone disease, contributing to 14-30% of cholecystectomies [4]. Symptomatic cholelithiasis also increases the risk for other complications. Around 3% of asymptomatic patients become symptomatic every year [5].

## Review

Choledocholithiasis

Choledocholithiasis is a clinical entity defined as the presence of stones in the CBD. It occurs in 10% of patients with symptomatic gallstones and 15% of patients with acute cholecystitis [6]. 95% of cases occur in patients with underlying cholelithiasis, whereas in 5% of cases, the stones arise de novo. Cholesterol stones are formed in the gallbladder only, while brown pigment stones can arise de novo via bacterial action on phospholipid and bilirubin in bile. The latter is seen in patients with hepatolithiasis, recurrent pyogenic cholangitis, and following endoscopic sphincterotomy. Black pigment stones are rare, formed in the gallbladder, and are seen in old age, hemolysis, alcoholism, and cirrhosis [7-9]. Stones are usually seen in distal one-third of CBD usually leading to the obstruction of the bile duct, thereby, raising bile pressure proximally and causing the duct to dilate. Normal pressure in the bile ducts is 10 to 15 cm water. The pressure of more than 15 cm of water decreases bile flow, while at the pressure of more than 25 cm of water, bile flow stops [9].

Around 45% of patients with choledocholithiasis remain asymptomatic for years, as stones can frequently pass silently into the duodenum. However, when they do cause symptoms, they tend to manifest as life-threatening complications such as cholangitis (fever, jaundice, or abdominal pain) and acute pancreatitis (fever, pain in the abdomen, and vomiting). Therefore, the discovery of choledocholithiasis should be followed by an intervention to remove the stones. Acute obstruction presents as pain, jaundice, and cholangitis, while chronic obstruction presents as pruritis and jaundice. Long-standing obstruction can lead to secondary biliary cirrhosis [10].

Courvoisier’s law states that if the gall bladder is palpable on clinical examination in a jaundiced patient, it is seldom due to gallstones [10]. Therefore, palpable gall bladder in jaundiced patients raises concern for malignancy and shall be investigated extensively. However, double impaction of CBD stone, Mirizzi syndrome, and others are a few notable exceptions to this law. Laboratory parameters have a high negative predictive value but a poor positive predictive value. Serum bilirubin is raised, usually between 2 and 5 mg/dL but rarely above 12 mg/dL. The rise is proportional to the extent of obstruction. Aspartate transaminase (AST) and alanine transaminase (ALT) show transient elevations and persistent elevations signal passage of stone(s) into duodenum, cholangitis, and pancreatitis. Alkaline phosphatase (ALP) is raised with the rise being more rapid, preceding that of bilirubin. However, the rise of ALP shows no relation with the extent of obstruction. Gamma-glutamyl transaminase (GGT) is the most commonly raised enzyme [11,12].

**Figure 2:**
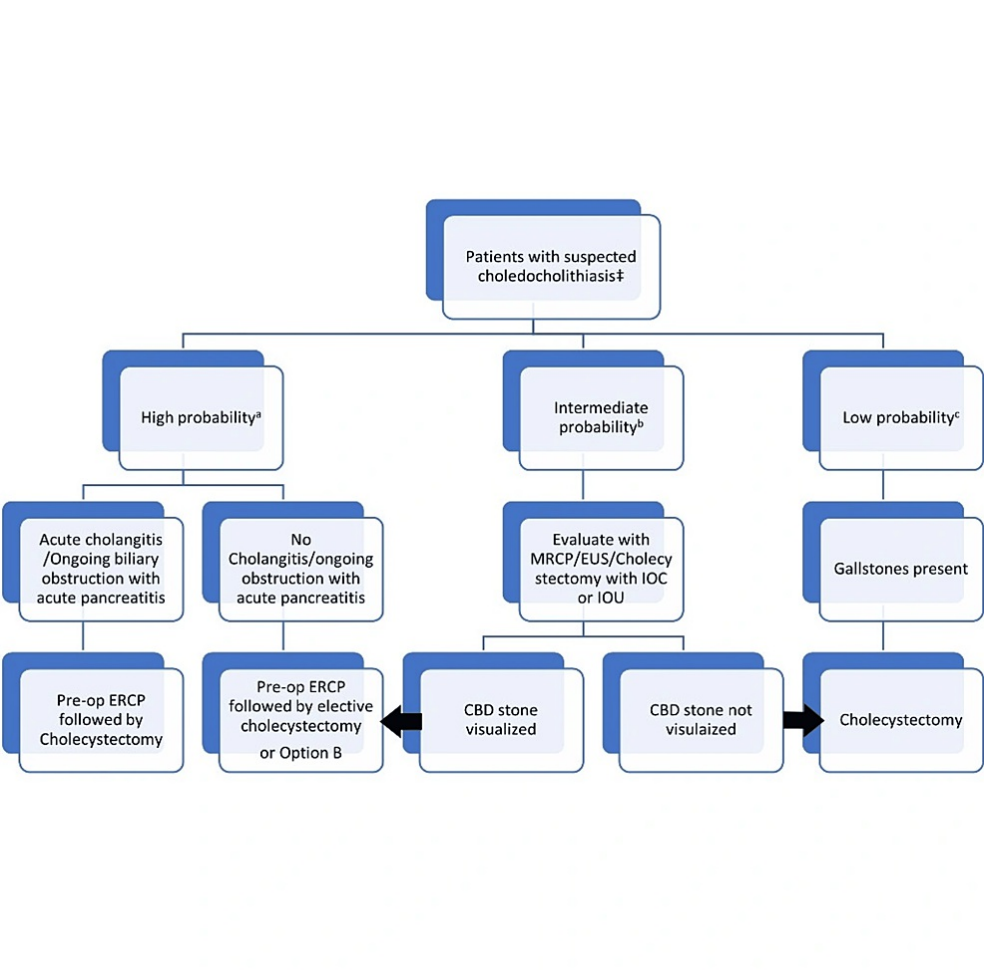
Approach to a patient with choledocholithiasis ^‡^Based on history and lab investigations. ^a^Any one of the following: CBD stone on abdominal US or cross-sectional imaging, acute cholangitis, serum bilirubin >4mg/dL, and a dilated CBD on US. ^b^Age >55 years, abnormal LFT, dilated CBD on abdominal US, or cross-sectional imaging. ^c^Gallstones or sludge visualized on abdominal US or cross-sectional imaging. Option B: Cholecystectomy with intraoperative CBD exploration or intraoperative ERCP or postoperative ERCP. CBD: common bile duct, ERCP: endoscopic retrograde cholangiopancreatography, EUS: endoscopic ultrasound, IOC: intraoperative cholangiography, IUS: intraoperative ultrasound, MRCP: magnetic resonance cholangiopancreatography Image Credit: Author Goyal MK

Diagnosis rests upon imaging. Ultrasound (US) can detect stones, and CBD dilatation (CBD >6 mm in those with gallbladder in situ and > 8 mm in those with cholecystectomy) is the initial imaging of choice with a sensitivity of 20-90% and a specificity of around 90%. However, it is poor for distal stones, which are obscured by gas. In patients who have had recurrent bouts of cholangitis, the bile duct may become fibrotic and is unable to dilate. Moreover, dilatation of the duct is sometimes absent in patients with choledocholithiasis because the obstruction is low-grade and intermittent. Magnetic resonance cholangiopancreatography (MRCP) is time-consuming and can miss stones in distal CBD and stones less than 5 mm in size, but it has a sensitivity of around 87% and a specificity of around 92% [13]. Endoscopic ultrasound (EUS) with a sensitivity of around 97% and a specificity of around 90% visualizes CBD more accurately but is invasive and requires expertise with limited availability [14]. Endoscopic retrograde cholangiopancreatography (ERCP) with a sensitivity of 80-93% and a specificity of 99-100% is both diagnostic and therapeutic but is invasive and is associated with multiple complications [15,16]. Percutaneous transhepatic cholangiography (THC) is an alternative when ERCP is unavailable but requires intrahepatic biliary radicals dilatation (IHBRD). Intraoperative cholangiography (IOC) and intraoperative US are alternative investigations that can be used when choledocholithiasis is diagnosed intraoperatively. The approach to a patient with choledocholithiasis is described in Figure 2.

Laparoscopic cholecystectomy with intraoperative bile duct exploration is being recognized as the procedure of choice in patients diagnosed with choledocholithiasis pre-operatively, avoiding the adverse events associated with ERCP [15]. Choledocholithiasis detected intraoperatively can be treated by either laparoscopic bile duct exploration, post-op ERCP, or conversion to an open cholecystectomy with bile duct exploration [15].

Difficult CBD stones present a significant challenge in gastroenterology, defined by factors such as the patient's clinical condition, the stone's characteristics (size >1 cm, >3 stones, stones proximal to biliary stricture, intrahepatic stones, and impacted stones), and anatomical considerations (surgically altered anatomy (SAA) and tortuous bile duct). The management of these stones requires a nuanced approach, often involving advanced endoscopic techniques beyond standard ERCP. The primary strategies for managing difficult CBD stones include EST, endoscopic papillary large balloon dilation (EPLBD), and mechanical lithotripsy (ML), with the choice of technique tailored to the patient's specific conditions and anatomical features [17]. EST and EPLBD are recommended as first-line therapies due to their effectiveness in reducing the need for ML, which is reserved for cases where initial attempts fail. Cholangioscopy-assisted lithotripsy, either electrohydraulic (EHL) or laser lithotripsy (LL), emerges as a valuable alternative for stones that are not amenable to extraction by EST, EPLBD, or ML, offering high success rates in stone clearance. For patients with SAA, such as those with Billroth II or Roux-en-Y gastric bypass, the management becomes even more complex. Techniques like balloon-assisted enteroscopy (BAE) and interventional endoscopic ultrasonography (I-EUS) have been developed to address these challenges, with I-EUS showing promise as a first-line treatment in expert hands due to its efficacy and low risk of adverse events [18]. Recent advancements also include the use of percutaneous cholangioscopy and EUS-guided approaches as novel methods for managing difficult CBD stones in patients with SAA. These techniques allow for direct visualization and treatment of stones, potentially reducing the need for multiple, invasive procedures. The management of difficult CBD stones in specific populations, such as post-liver transplantation patients or those who have undergone weight loss surgery, requires specialized approaches due to the unique anatomical and physiological changes in these groups. Techniques like EUS-directed transgastric ERCP (EDGE) and laparoscopic-assisted transgastric ERCP (LA-ERCP) have been developed to address these needs, offering high success rates [18].

Acute cholangitis

Acute cholangitis is the most serious and lethal complication of cholelithiasis. 28% to 70% of cases of cholangitis occur due to gallstones [19]. Diagnosing in the early stages is difficult as clinical features are often absent or non-specific. Pus under pressure in the bile ducts leads to septicemia. Bile duct obstruction is necessary but not sufficient to cause cholangitis. The presence of bacteria is a must for pathogenesis with the most commonly implicated organisms being E. coli, Klebsiella, Pseudomonas, Proteus, and Enterococci. Anaerobes are present in 15% of cases.

The classical combination of fever, jaundice, and right upper quadrant pain (Charcot’s triad) is present in only 50-75% of cases. Reynolds’ pentad (altered mental status with hypotension plus Charcot’s triad) is seen in even fewer cases. Older patients usually present with lethargy, confusion, or delirium. Complications can occur in untreated cases and include cholangitic abscess, sepsis, and multi-organ dysfunction. The most common clinical feature is high-grade fever (>102°F), seen in 95% of cases, followed by right upper quadrant tenderness (90% of cases) and jaundice (80% of cases). The most common laboratory parameters supporting a diagnosis are raised bilirubin, raised ALP/GGT, and neutrophilic leukocytosis. A blood culture is positive in most. Concomitantly raised lipase is suggestive of pancreatitis. Persistent transaminitis is indicative of cholangitic hepatic abscess [20]. The severity of the disease is assessed based on Tokyo guidelines, which classify patients into those with severe disease (at least one organ dysfunction), moderate disease (any two of abnormal WBC count/high-grade fever/age ≥75 years/bilirubin ≥5 mg/dL/hypoalbuminemia), and mild disease (not fulfilling above criteria).

Diagnosis of cholangitis relies upon clinical, laboratory, and imaging findings. It requires the presence of either one of fever (with or without chills) or raised WBC count/neutrophils and CRP, with laboratory features suggestive of cholestasis (ALP or GGT >1.5 times upper limit of normal plus serum bilirubin ≥2 mg/dL) and imaging evidence of choledocholithiasis [21].

Treatment consists of correcting fluid and electrolyte disturbances, pain control, empirical antibiotics after drawing blood cultures (as soon as possible) later switching on as per need, and monitoring for organ dysfunction. Antibiotics are continued for four to five days after control of infection. Patients are assessed within six to 12 hours. Those deteriorating or those with severe disease at presentation should undergo immediate decompression with ERCP being the method of choice. EUS can be tried when ERCP fails or is not feasible. Percutaneous or surgical drainage is indicated if endoscopy is not feasible. In stable patients, decompression can be done within 24 to 48 hours. Cholecystectomy is done after biliary drainage in mild or moderate cases. In contrast, in severe cases it should be done after improvement of the general condition or after an interval of four to six weeks [22].

Acute cholecystitis

Acute cholecystitis is the most common complication of cholelithiasis, with 90% of cases of cholecystitis being due to gallstones. It typically develops in symptomatic patients and is considered a disease of young and healthy women with a favorable prognosis [23].

It occurs as a result of chronic obstruction of bile resulting in damage to the gallbladder mucosa and release of intracellular enzymes with activation of inflammatory mediators like phospholipase A, which converts lecithin to lysolecithin implicated in the pathogenesis of disease by increasing protein secretion, decreasing water reabsorption, promoting leukocyte invasion, and increasing PG-E and F1α levels. Enteric bacteria can be cultured from gallbladder bile but are not believed to trigger the onset of acute cholecystitis [24].

Pathological features observed during the evolution of the disease are a distended gallbladder with inflammatory exudate and, rarely, pus over the first few days. Later on, bile pigments are absorbed and replaced by thin mucoid fluid, pus, or blood. In untreated cases over the long term, an overdistended gallbladder lumen filled with clear mucoid fluid (hydrops) is seen due to the absence of bile entry into the gallbladder and absorption of all the bilirubin within it.

The most common symptoms observed are biliary pain lasting for more than six hours, with a prior history of pain present in around 75% of cases. Fever is a common symptom and is usually low to moderate grade. High-grade fever should raise suspicion of gangrene or perforation. Jaundice is seen in only 20% to 40% of cases and is usually less than 4 mg/dL. A serum bilirubin of >4mg/dL in a patient with acute cholecystitis suggests the presence of choledocholithiasis or Mirizzi syndrome. The most common sign is right subcostal tenderness, with the presence of Murphy’s sign in most (inspiratory cessation on palpating the right subcostal area with deep inspiration). Sonographic Murphy’s sign has a positive predictive of 90%. A palpable gallbladder is felt in 30% of cases. The pain of untreated acute cholecystitis generally resolves in seven to 10 days. Not uncommonly, symptoms remit within 48 hours of hospitalization and the episode resolves without complications in about 83% of patients. Gangrenous cholecystitis is seen in around 7% of cases, gallbladder empyema in around 6%, perforation in around 3%, and emphysematous cholecystitis in 1% to 4% of cases [25].

The usual laboratory features consist of leukocytosis with a shift to immature neutrophils with often mild elevations in serum aminotransferase and ALP levels. Serum bilirubin of more than 4 mg/dL signifies the presence of CBD obstruction while pancreatitis should be suspected if serum amylase is more than 1000IU/L. Empyema or perforation should be considered if WBC count is more than 15,000/mm^3^, in the setting of worsening pain, high fever (temperature >102°F), and chills. US abdomen is the single most useful imaging study in acutely ill patients and can identify gallstones, gallbladder wall thickening (>4 mm), pericholecystic fluid, and gallbladder enlargement (long axis ≥8 cm, short axis ≥4 cm) [26]. MRI/MRCP is useful for diagnosing acute cholecystitis and is recommended if the abdominal US does not provide a definitive diagnosis. CT abdomen helps detect complications and exclude alternate causes. It is not warranted in obvious cases but is useful when diagnosis or optimal timing of surgery is in doubt [22].

Diagnostic criteria and severity of disease are based on Tokyo guidelines. Diagnosis depends on the presence of local and systemic signs of inflammation along with imaging findings characteristic of the disease. Severe cases have at least one organ dysfunction, while moderate cases have any one of abnormal WBC count (>18,000/mm^3^), a palpable tender mass in the right upper quadrant, duration of symptoms more than 72 hours, and presence of marked local inflammation. Mild disease does not have any of the aforementioned features [27].

**Figure 3:**
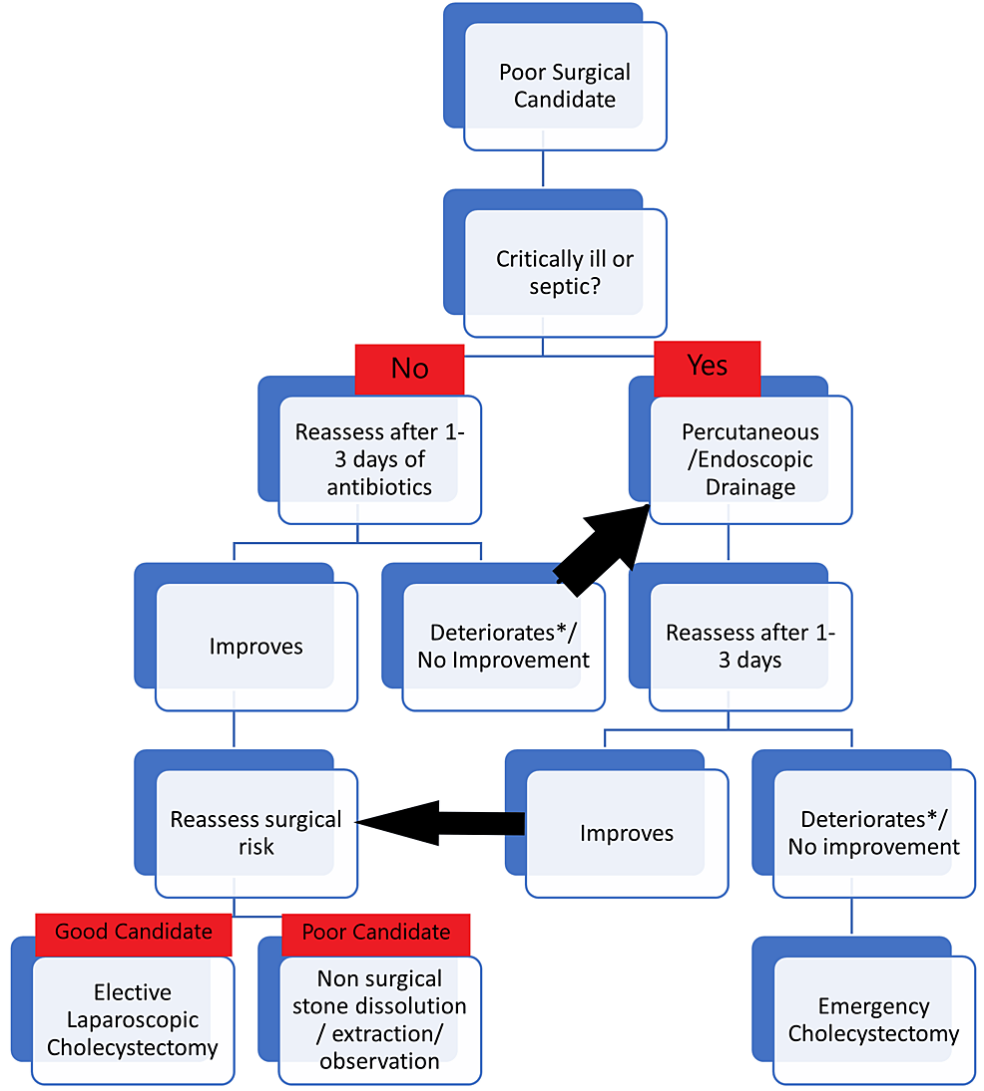
Approach to poor surgical candidates with acute calcular cholecystitis *Deteriorates at any time during the period of assessment. Image Credit: Author Goyal MK

Treatment consists of correcting fluid and electrolyte imbalances, controlling pain, keeping the patient nil per oral with placement of a nasogastric tube in case of abdominal distention or persistent vomiting and antibiotic therapy (to continue till cholecystectomy in mild to moderate cases or till clinical resolution of cholecystitis in severe or complicated cases) [28]. Emergency cholecystectomy is indicated in case of gangrene/necrosis of the gallbladder, perforation of the gallbladder, emphysematous cholecystitis, intractable pain, and progressive signs and symptoms. In all other cases, the disease can be stratified based on Tokyo or AASA staging systems. Tokyo guidelines recommend same admission laparoscopic cholecystectomy for all cases irrespective of severity, while WSES guidelines recommend interval cholecystectomy in critical patients (septic shock or contraindication to anesthesia) [27]. Early cholecystectomy (within three to seven days of admission or 10 days of symptom onset) has better outcomes and lower costs. The management of poor surgical candidates is explained in Figure 3.

Endoscopic drainage can be done via transpapillary (ERCP) or transmural (EUS) routes, with the technical success and clinical efficacy of the latter being better across various studies [29,30].

Emphysematous cholecystitis

It is a severe form of acute cholecystitis seen in 1% to 4% of cases, characterized by infection with gas-forming organisms like Clostridium welchii. It is usually more common in older males, with diabetes mellitus and coronary artery disease, and presents with trivial symptoms. It progresses to a rapidly deteriorating condition if left untreated. It can be suspected in cases with crepitus adjacent to the gallbladder or the presence of unconjugated jaundice as a result of hemolysis caused by clostridial infection. It often heralds the development of gangrene and perforation. It can be categorized into three stages (AAST classification). Stage 1 is characterized by the presence of gas in the gallbladder lumen (only in some parts) while in stage 2, there is gas in the entire gallbladder wall. Stage 3 is diagnosed by the presence of gas in pericholecystic fluid and within adjacent tissues and indicates gangrene/perforation [31]. The abdominal US may erroneously report gas obscuring the gallbladder, which reflects air in the wall of the gallbladder. The best imaging modality for diagnosis is CT abdomen. MRI shows the presence of intramural air and necrosis. Treatment is emergency antibiotic therapy with anaerobic coverage and emergency cholecystectomy.

Cholecystoenteric fistula

Cholecystoenteric fistula occurs in around 2% to 3% of patients with cholelithiasis, with gallstones eroding through the neck of the gallbladder. It is a rare and complex complication of cholelithiasis, characterized by an abnormal connection between the gallbladder and the gastrointestinal tract. It predominantly affects women in their 60s and 70s, with the incidence rate among women being 1.9 to 2.5 times higher than in men. Additional predisposing factors include peptic ulcer disease, biliary and gastrointestinal malignancies, inflammatory bowel disease, right-sided diverticulitis, previous abdominal trauma, and surgical interventions. Duodenum is the most common fistula entry point (70% cases), followed by hepatic flexure of the colon (8% to 26% cases), and rarely the stomach and duodenum. It can be occasionally asymptomatic as stones can pass through, while in others it can present like acute cholecystitis. In certain instances, patients may present with gastrointestinal bleeding symptoms. Cholangitis does not occur as the biliary tract is already decompressed. Bile acid diarrhea can occur in cholecystocolonic fistula. It should be suspected in the presence of pneumonia and in elderly patients with a history of cholelithiasis and diverticulosis who present with bowel obstruction. Diagnosis is confirmed by barium contrast studies or using ERCP. However, occasionally it is not identified until surgery [32].

Gallstone ileus

Gallstone ileus occurs when a gallstone of size more than 2.5 cm is impacted in the bowel via cholecystoenteric fistula or following sphincterotomy. The term is a misnomer as the impaction leads to actual obstruction and is responsible for less than 0.5% of cases of small bowel obstruction. The most common site of impaction is the distal ileum, around 2 feet proximal to the IC valve. It is most commonly seen in older adults, and diagnosis is often delayed [33]. Bouveret syndrome refers to gastric outlet obstruction from duodenal impaction of a large gallstone that has migrated through a cholecystoduodenal fistula. Symptoms consist of episodic pain, vomiting, and obstipation, associated with abdominal distention. Jaundice is seen in less than 15% of cases. Laboratory parameters can reveal leukocytosis, electrolyte disturbances, and transaminitis. Diagnosis is suspected based on the presence of Rigler’s triad on abdominal x-ray (presence of pneumobilia, dilated small bowel loops, and presence of a large stone distal to it) and confirmed with a CT abdomen. Endoscopy is useful for diagnosing Bouveret syndrome [34]. Treatment consists of surgery, which comprises enterolithotomy, cholecystectomy, and fistula closure. Surgery can be done in a single step in low-risk patients (ASA I/II) or in two steps (enterolithotomy followed by cholecystectomy and fistula closure) in high-risk patients (ASA III/IV). Non-surgical options include endoscopic or extracorporeal lithotripsy and can be done in patients who are not suitable candidates for surgery [33].

Mirizzi syndrome

Mirizzi syndrome refers to common hepatic duct obstruction due to extrinsic compression from an impacted stone in the cystic duct or infundibulum of the gallbladder. It is seen in around 0.05-4% of specimens of cholecystectomies and is more common in women (50% to 77% cases) owing to the higher incidence of cholelithiasis in them. It is associated with carcinoma gallbladder in 5% to 28% of cases across different studies [35]. Right upper quadrant pain is the most common symptom, seen in 54% to 100% of patients. Other presentations are jaundice (seen in 24% to 100% of cases) and Charcot’s triad (seen in 44% to 71% of cases). Acute cholecystitis is seen in 1/3rd cases while acute pancreatitis is rare. Laboratory investigations reveal raised bilirubin in 90% of cases. Leukocytosis is usually seen in complicated cases. Diagnosis should be suspected in all cases of cholangitis and gallstone ileus and patients with difficult cholecystectomy. Confirmation of diagnosis usually relies on imaging. Abdominal US is the initial investigation in most cases but has a sensitivity of only 23% to 46%. CT abdomen with a sensitivity of 30% to 50% is only helpful in ruling out malignant obstruction causes. MRCP is the most sensitive investigation (80-90%) and reveals the extent of inflammation. MRCP has classically described the Meniscus sign for this entity. ERCP has both diagnostic and therapeutic roles [36]. The diagnostic features on imaging include dilatation of the biliary system above the gallbladder neck, the presence of an impacted stone on the gallbladder neck, and an abrupt change to the normal diameter of the common duct below the level of the stone. Surgery is the mainstay of treatment with pre-operative ERCP and stenting the preferred approach. Decompression using ERCP can be done in patients not fit for surgery. Mirizzi syndrome is classified into five types based on Csendes classification. Various types along with surgical options are summarized in Table 1 [37,38]. Moreover, laparoscopic cholecystectomy has almost replaced the open technique for routine cholecystectomies since the early 1990s [39].

**Table 1:** Mirizzi syndrome classification with surgical options CBD: common bile duct, CC-D: cholecystoduodenostomy, CD-D: choledochoduodenostomy, CD-J: choledochojejunostomy

Type	Description	Surgery
I	External compression of bile duct	Partial or total cholecystectomy with or without CBD exploration
II	Cholecystobiliary fistula - up to 1/3 of the bile duct wall erosion	Cholecystectomy with closure of fistula or choledochoplasty with remnant gallbladder
III	Cholecystobiliary fistula - up to 2/3 of the bile duct wall erosion	Choledochoplasty or bilioenteric anastomosis (CD-D/CC-D/CD-J)
IV	Cholecystobiliary fistula - complete destruction of the bile duct wall and fusion with gallbladder	Bilioenteric anastomosis (choledochojejunostomy)
Va	Cholecystoenteric fistula	Enterolithotomy plus cholecystectomy plus fistula closure
Vb	Cholecystoenteric fistula with gallstone ileus	Enterolithotomy plus cholecystectomy plus fistula closure

## Conclusions

Cholelithiasis is one of the most common diseases worldwide with a high risk of complications in symptomatic patients. These complications lead to significant morbidity and in some cases mortality. Early detection and appropriate management of these complications is pivotal as it can prevent the significant burden on patients and health care institutes. The diagnosis and treatment options for various complications are ever-evolving. A multidisciplinary approach involving gastroenterologists, radiologists, surgeons, anesthesiologists, and an intensive care team is required in most cases with cholecystectomy being the pivotal step in management.
